# A Review of the Ethnobotanical Use, Chemistry and Pharmacological Activities of Constituents Derived from the Plant Genus *Geijera* (*Rutaceae*)

**DOI:** 10.3390/metabo14020081

**Published:** 2024-01-23

**Authors:** Deepika Dugan, Rachael J. Bell, Robert Brkljača, Colin Rix, Sylvia Urban

**Affiliations:** 1 Marine and Terrestrial Natural Product (MATNAP) Research Group, School of Science (Applied Chemistry and Environmental Science), RMIT University, GPO Box 2476, Melbourne, VIC 3001, Australia; s3646443@student.rmit.edu.au (D.D.); rbell0030@gmail.com (R.J.B.); colin.rix@rmit.edu.au (C.R.); 2 Monash Biomedical Imaging, Monash University, Clayton, VIC 3168, Australia; robert.brkljaca@monash.edu

**Keywords:** *Geijera*, wilga, biological activity, pharmacology, customary use, analgesic, toothache, anti-cancer

## Abstract

*Geijera* Schott is a plant genus of the *Rutaceae* Juss. (rue and citrus) family, comprising six species which are all native to Oceania. Of the plants belonging to this genus, the most significant species that has a customary use is *Geijera parviflora*, which was used by Indigenous Australians, primarily as a pain reliever. Herein, a comprehensive review of the literature published on the genus *Geijera* from 1930 to 2023 was conducted. This is the first review for this plant genus, and it highlights the chemical constituents reported to date, together with the range of pharmacological properties described from the various species and different parts of the plant. These properties include anti-inflammatory, anti-microbial, anti-parasitic, insect repellent, analgesic, neuroactive, and anti-cancer activities. Finally, a reflection on some of the important areas for future focused studies of this plant genus is provided.

## 1. Introduction

The genus *Geijera* contains six accepted species that are native to Australia, New Guinea, and New Caledonia [1]. Although the International Plant Names Index has over twenty species names listed in association with this genus, a large proportion of these are either synonyms of the accepted species or names that have been superseded due to taxonomic reclassification. The six species of the genus are listed below in Table 1 [2,3]. The species of this genus are found in rainforests, dry rainforests, woodlands, dry scrub, and open inland areas [4].

To date, phytochemical investigations have only been conducted on four of the six *Geijera* species. The chemical constituents provided in this review are, therefore, limited to the studies conducted on *Geijera parviflora* Lindl., *Geijera linearifolia* (DC.) J.M. Black (both endemic to Australia), *Geijera salicifolia* Schott (endemic to Australia, Papua New Guinea, and New Caledonia) and *Geijera balansae* (Baill.) Schinz & Guillaumin (endemic to New Caledonia). No phytochemical information is available for *Geijera cauliflora* Baill., and *Geijera tartarea* T.G. Hartley ex Munzinger & Bruy, which are both endemic to New Caledonia. Phytochemical investigation of the latter two species has been neglected possibly due to their rarity, their inaccessibility, or that they occur in a remote location. *Geijera tartarea* is a newly described, rare, and endangered species [5]. The 117 reported compounds in this review have been grouped on the basis of their chemical class and are numbered sequentially in Table 2, Table 3, Table 4 and Table 5.

**Figure 1:**
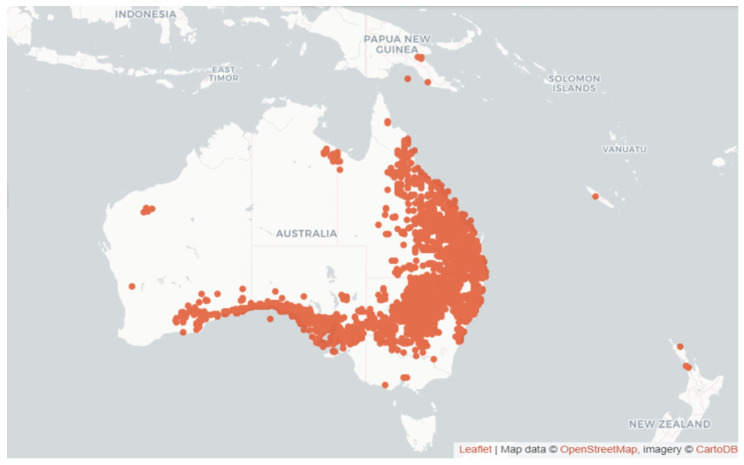
Occurrence of species from the genus *Geijera* Schott [3].

The flowering plants of most endemic *Rutaceae* species in Oceania occur as low scleromorphic shrubs, whereas all species of *Geijera* can be described as large sclerophyllous shrubs [6]. Figure 1 illustrates the geographical distribution of *Geijera* species in Oceania.

The customary use of plants has been occurring for at least 65,000 years by the Indigenous Australians [7]. Interest in the chemical constituents of the *Geijera* species, particularly *G. parviflora*, has been motivated by the customary use of this plant in Australian bush medicine. Commonly known as dogwood or wilga (‘Wilgarr’ in the Wiradjuri language, ‘Nhiitaka’ or ‘Katha’ in Paakantyi (Barkindji), ‘Puri’ or ‘Buri’ in Mutthi Mutthi and ‘Dhiil’ or ‘Dheal’ in the Gamilarray, Yuwaalaray and Yuwaalayaay language groups), *G. parviflora* is considered a sacred tree and is of cultural importance to the Indigenous people of Australia, especially during burials and ceremonies [8]. It is a hardy, long-lived species that grows about 8 m tall with a wide, dense canopy and it can live for over 100 years.

The leaves of *G. parviflora* were used to prepare a ceremonial smoke together with leaves from other trees such as ‘Badha’ or ‘Budda’ Native Sandalwood, *Eremophila mitchellii* Benth., ‘Coolabah’ Eucalyptus coolabah Blakely & Jacobs and ‘Gurraay’ White Cypress Pine *Callitris columellaris* F.Muell. [8]. *G. parviflora* leaves were also used in ceremonies where they were baked, powdered, and smoked with other plant materials to induce intoxication and drowsiness, akin to the effects of alcohol [9]. The leaves were also chewed or placed into dental cavities for the relief of toothache or crushed and used as an external pain reliever [10]. An infusion of the leaves was used both internally and externally to relieve pain [11,12]. Leaf infusions were also used for bathing to provide skin care and to relieve sore muscles; they were used cold for sore eyes and ears, and apart from this, they were drunk to cure blood disorders [13]. The hot leaves were used as a poultice on sores and boils, and the bark was steeped in water for use as a laxative [13]. The leaves were also burned around camp sites as an insect repellent and the wood was used for spears and boomerangs [13]. Apart from these uses, the tree was valued as a source of pollen and nectar for honey production, the fruits and flowers of *G. parviflora* were eaten, and its aromatic leaves were used during cooking as a flavoring for emu meat [14,15].

*G. parviflora* is the most extensively studied species of the genus *Geijera* and it exhibits considerable variability in the composition of its essential oils and other chemical constituents, such as its coumarins [16]. During an initial investigation by Penfold in 1930, he noted that morphologically indistinguishable specimens had different chemotypes based on the character of their leaf essential oils [17,18,19]. The leaves of *G. parviflora* were observed to exhibit selective palatability as fodder for sheep, wherein certain plants would be readily consumed by stock whilst others would not. Two coumarins, dehydrogeijerin **13** and geiparvarin **2** (Table 2), were later isolated by Lahey and Macleod from specimens deemed either ‘unpalatable’ or ‘readily eaten’ [20]. It was found that **13** was only present in the unpalatable variety, and that **2** was only in the readily eaten variety. It was also observed that the readily eaten variety ‘Tree wilga’, occurred in drier areas, while the variety deemed unpalatable, ‘Lavender bush’ occurred in areas with greater than 500 mm of rainfall per annum [16]. The connection that was drawn between the two different coumarins present in the specimens and their palatability to sheep has not been validated [21]. After further research conducted by Brophy and Goldsack [16], *G. parviflora* now has four established chemotypes, based upon differing compositions of the leaf essential oils of the plant, with another four possible chemotypes having been tentatively identified by Sadgrove et al. [16,22].

*G. salicifolia* (scrub wilga, greenheart, green satinheart) is a long-lived, drought-tolerant and hardy species, utilised mainly for its timber, which was used to make fishing rods and cabinetry [23]. Its wood was traditionally used for making implements, weapons, and jewellery [24]. According to the Dharawal pharmacopeia collection recorded by Auntie Frances Bodkin, a Dharawal elder, *G. salicifolia* is also commonly called wilga and (similarly to *G. parviflora*) its leaves have customary use by the Dharawal people for pain relief, whereby they are chewed to alleviate toothache [24]. The vapors from the hot leaves are also used to relieve headache [24]. Two chemotypes of this species have been identified based upon differing composition of its leaf essential oils [16].

Uses of the other species from the *Geijera* genus for medicinal or other purposes (apart from timber) have not been recorded.

The purpose of this study was to review the chemical constituents within the genus *Geijera* Schott as reported up to December 2023, this being the first such review on the genus. *Geijera* is a genus that belongs to the family *Rutaceae* Juss. (rue and citrus family), which contains about 2100 species in 154 genera [25]. Apart from providing important nutritional benefits, many members of the *Rutaceae* are valuable sources of bioactive compounds such as alkaloids, coumarins (notably furano- and pyrano-coumarins), volatile oils, flavonoids, and limonoids [26]. This review provides a detailed account of the chemical constituents reported from the *Geijera* genus to date. However, it is only representative of four of the six *Geijera* species, as no studies have been reported for the chemical constituents of *G. cauliflora* and *G. tartarea*. In addition to the description of the constituents isolated from *Geijera*, their reported pharmacological activities are also summarised. Although a detailed treatment of the various specific pharmacological activities reported from these compounds is beyond the scope of this review, a concise summary has been provided. Specifically, the pharmacological activities relevant to the traditional use of *G. parviflora* are summarised to aid identification of constituents, which might be responsible for the customary medicinal uses of *G. parviflora* in Australian bush medicine. Constituents with notable properties such as anti-cancer activity are also included. Subsequent database searching of the relevant chemical constituents provided an account of what main types of pharmacological activities have been reported in the literature. Therefore, the documented pharmacological activities in this review are not necessarily reported from within the same publications that identified the chemical constituents in the *Geijera* plant species.

## 2. Methodology

The scientific literature published on the genus *Geijera* from 1930 to 2023 has been reviewed with a particular focus on publications pertaining to the phytochemicals that are specific to this genus. The databases employed for compiling this review included Google, Google Scholar, ScienceDirect (147 results), SciFinder^n^ (50 results), Scopus (44 results), Springer Link (78 results), PubMed (12 results), Wiley (124 results), and the Web of Science (43 results). Within the results, there were approximately 20 publications specifically reporting the isolation of chemical constituents from this plant genus. The search terms or keywords included *Geijera*, *Geijera parviflora*, *Geijera salicifolia*, *Geijera linearifolia*, *Geijera balansae*, *Geijera cauliflora*, *Geijera tartarea*, and wilga.

## 3. Chemical Constituents in *Geijera* Species

A total of 117 plant compounds have been identified via phytochemical investigations of four plant species of the *Geijera* genus, covering *G. balansae*, *G. linearifolia*, *G. parviflora*, and *G. salicifolia*. The compounds can be generally assigned to the following classes: coumarins, alkaloids, phenolic compounds, a flavonoid, fatty alcohol esters, fatty acid esters, phenylpropanoids, terpenes and terpenoids, and these appear sequentially in Table 2, Table 3, Table 4 and Table 5.

**Figure 2:**
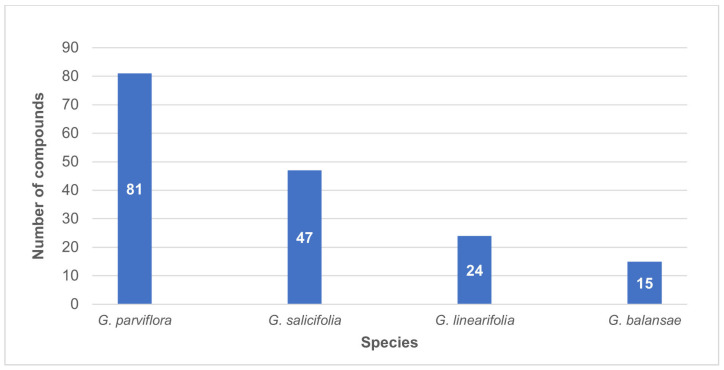
Total plant compounds identified to date from each of the studied *Geijera* species.

Most of the compounds identified from the genus *Geijera* originate from *G. parviflora*, which has been studied more than the other species, mainly due to its traditional medicinal uses by Indigenous cultures, as well as its utility as stock fodder during times of drought in the early- to mid-20th century. As shown in Figure 2, the other *Geijera* species have had little study in comparison. Hence, there is clear potential for further compound identification and discovery, especially considering the various bioactivities displayed by the chemical constituents identified to date. It also shows that while 117 compounds have been identified among the four species, many of these occur across multiple species of *Geijera*.

**Figure 3:**
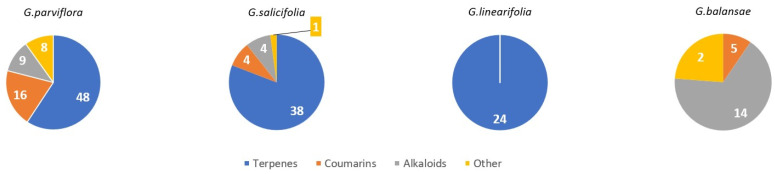
Distribution of plant compounds identified to date within *Geijera* species. The number denotes the number of compounds of each class within the *Geijera* species.

The number of chemical constituents according to the compound classes identified from the four *Geijera* species studied is illustrated in Figure 3, showing that the terpenes represent by far the most frequently isolated compound class across these species. It also shows that *G. balansae* is the only species to not have any terpenes reported. Terpenes are dominant components in the plant kingdom, so it is unlikely that *G. balansae* does not contain terpenes, but rather is indicative that no terpenes have yet been reported because the extraction methods employed to study this species to date have specifically targeted the isolation of alkaloids [27,28]. Similarly with *G. linearifolia*, only terpenes have been reported; however, this does not suggest only terpene-like compounds are being produced by *G. linearifolia*, but that further study of *G. linearifolia* is needed to reveal additional compound classes present.

Investigations on the other three *Geijera* species have focused on the plant essential oils which were obtained via hydro-distillation, as well as other targeted extraction methods employed for the extraction of coumarins and alkaloids. Studies on *G. linearifolia* reported the presence of terpenes, but not any other compound classes because only the volatile component/essential oil from this species has been studied [16].

As a result of conducting this review, it became evident that more than 60% of the compounds that have been reported from this plant genus require further verification and validation using spectroscopic techniques and other isolation strategies. Several compounds (largely terpenes and terpenoids) were identified solely based on GC-MS retention times, molecular weights, and database comparison, which can be inadequate for the elucidation of geometric/structural/stereoisomeric structures. The formation of artefacts that can result from isolation procedures, where plant materials are subjected to thermal treatment during hydro-distillation and gas chromatography, is another consideration to bear in mind. An example of this is the sesquiterpene geijerene **70**, which is accepted to be a thermal Cope rearrangement product that is formed from its precursor, pregeijerene **68**, which is a major constituent of the essential oil of one *G. parviflora* chemotype [22,29].

Despite considerations like the ones stated above, most of the reported compounds have been included in this review due to the variety of pharmacological properties that they possess. Minor constituents present in less than 1% of the essential oils, as well as constituents of aged plant essential oils that have been reported via GC-MS analysis, were omitted from the review. The rationale for this was due to their insignificant quantity and/or high likelihood of them being artefacts formed by processes such as oxidation and polymerisation as the oils age over several months or years. An interesting comparison of the character of aged essential oils with fresh samples that was performed by Sadgrove et al., demonstrated that the antimicrobial activity of aged samples increased compared to that of the fresh samples [9].

### 3.1. Coumarins

Coumarins are common in the *Rutaceae* plant family, and they primarily act as phytoalexins and allelochemicals. Their physiological roles are diverse and include protection of the plant against traumatic injury, microorganism infection by inhibition of the growth of bacteria and fungi, facilitating iron uptake from soil, as well as providing protection by repelling herbivorous insects [30]. The *Rutaceae* produce a range of pyrano-, furano- and prenylated-coumarins, in addition to the simple coumarins, all of which frequently display potent pharmacological effects [31]. The activity of coumarins is often attributed to the reactivity of the benzofuran system in their molecular structure; however, many simple coumarins also possess potent activity, e.g., the toxicity of umbelliferone **1** (Table 2) against insect herbivores and rodents [31,32,33,34]. Amongst other functions, **1** is a key intermediate in prenylcoumarin biosynthesis, which gives rise to the furanocoumarins and pyranocoumarins such as angelicin (isopsoralen) **16** and xanthyletine **17** [35].

Within *Geijera*, coumarins have been identified in the leaves of *G. balansae*, *G. parviflora* and *G. salicifolia* as well as the bark of *G. balansae*. Nineteen coumarins have been reported from the genus, consisting of nine monosubstituted coumarins **1–9** including umbelliferone **1**, and six disubstituted coumarins **10–15**, furanocoumarin angelicin (isopsoralen) **16**, and three pyranocoumarins **17–19**. Compounds **11**, **12**, and **15–17** were identified by Sadgrove et al. in trace amounts, based on GC-MS analysis of extracts. The unequivocal identification of these five coumarins within *G. parviflora* requires further investigation using targeted extraction strategies, in conjunction with the application of spectroscopic techniques for structure identification/elucidation. Luvangentin **18** was isolated from the leaves and xanthoxyletin **19** was isolated from the bark of the New Caledonian species *G. balansae* by Mitaku et al. (Table 2).

The coumarins geiparvarin **2** and dehydrogeijerin **13** were isolated by Lahey and Macleod from *G. parviflora* specimens deemed either ‘readily eaten’ or ‘unpalatable’. It was found that **13** was only present in leaves of the unpalatable variety which occurs in wetter areas, and that **2** was only present in leaves of the readily eaten variety, which occurs in drier areas [16]. Further work is needed to establish the validity of the connection between the palatability of these two chemotypes and the coumarins present therein.

Geiparvarin **2** has been found only in *G. parviflora* and *G. salicifolia,* and being a major constituent of leaf extracts, it has been identified as one of the main contributors to the pharmacological activities of these plants [36]. Its derivatives, such as 2′,3′-dihydrogeiparvarin **6**, parvifloranine A **8**, parvifloranine B **9**, and 6-(methoxyl) geiparvarin **14**, have only been found in *G. parviflora* so far. This coumarin and its derivatives can be said to be chemotaxonomic markers for the two abovementioned *Geijera* species, although more phytochemical investigation is required to establish whether they are also present in the other species of the genus or elsewhere within other taxa.

**Table 2:** Coumarins identified within the genus *Geijera*.

Compound and Exact Mass (Da)	Source	Method of Identification	Reference	Pharmacological Activity of Compound (Various Sources)
**1** umbelliferone	*G. salicifolia* (leaves)	Melting point, IR and ^1^H NMR	[37]	Anti-inflammatory, antinociceptive, anti-hyperglycaemic, antibacterial, antifungal, inhibition of DPPH, hydroxyl, superoxide anion and ABTS radicals, molluscicide, antifeedant, anti-tumour, antimutagenic, fluorescent (sunscreen agent), bone-protective, anti-biofilm [38,39,40]
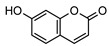				
162.0317				
**2** geiparvarin	*G. parviflora* (leaves)	Combustion analysis, chemical derivatisation, UV, IR (*G.p*)	[20,37]	Anti-cancer, monoamine oxidase B inhibitor [41,42,43]
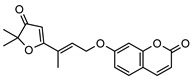				
326.1154	*G. salicifolia* (leaves)	IR and ^1^H NMR (*G.s*)		
**3** auraptene	*G. parviflora* (fruit/seeds)	IR and ^1^H NMR	[44]	Increases collagen I expression, anti-bacterial, anti-fungal, antileishmanial, anti-cancer, and antioxidant [45,46]
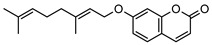				
298.1569				
**4** marmin	*G. parviflora* (fruit/seeds)	IR and ^1^H NMR	[44]	No significant anti-inflammatory activity [47]
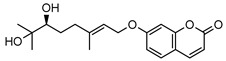				
332.1624				
**5** 6′-dehydromarmin	*G. parviflora* (fruit/seeds)	IR and ^1^H NMR	[44]	Anti-inflammatory, cytotoxic [10]
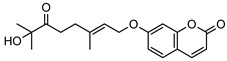				
330.1467				
**6** 2′,3′-dihydrogeiparvarin	*G. parviflora* (fruit/seeds)	IR and ^1^H NMR	[44,48]	Anti-cancer [48,49]
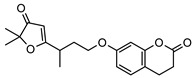	*G. salicifolia* (leaves)			
330.1467				
**7** (*R*)-6-O-(4-geranyloxy-2-hydroxy) cinnamoylmarmin	*G. parviflora* (leaves)	2D NMR	[10]	Cytotoxic, anti-inflammatory [10]
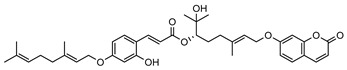				
630.3193				
**8** parvifloranine A	*G. parviflora* (leaves)	2D NMR, ECD and MS	[50]	Anti-inflammatory [50]
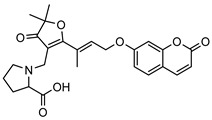				
453.1788				
**9** parvifloranine B	*G. parviflora* (leaves)	2D NMR, ECD and MS	[50]	No significant anti-inflammatory activity [50]
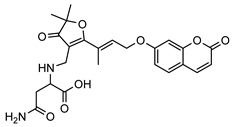				
470.1689				
**10** geijerin	*G. salicifolia* (bark)	Chemical derivatisation, UV, and IR	[37,51]	Acetylcholinesterase inhibitor [52]
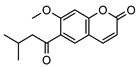	*G. parviflora* (leaves)	Melting point, IR and ^1^H NMR		
260.1049				
**11** scoparone	*G. parviflora* (leaves)	GC-MS	[21]	Antifungal, anti-inflammatory, antioxidant, anti-apoptotic, anti-fibrotic, and hypolipidemic [53,54]
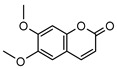				
206.0579				
**12** suberosin	*G. parviflora* (leaves)	GC-MS	[21]	Anti-inflammatory and anticoagulant [55,56]
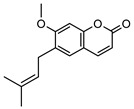				
244.1099				
**13** dehydrogeijerin	*G. parviflora* (leaves)	Chemical derivatisation, UV, and IR (*G.p*)	[20,37]	Anti-inflammatory activity, acetylcholinesterase inhibitor [52,57]
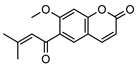	*G. salicifolia* (leaves)	IR and ^1^H NMR (*G.s*)		
258.0892				
**14** 6-(methoxyl) geiparvarin	*G. parviflora* (leaves)	^13^C and ^1^H NMR	[10]	Anti-inflammatory, cytotoxic [10]
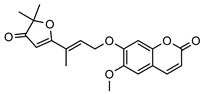				
356.1260				
**15** osthole	*G. parviflora* (leaves)	GC-MS	[22]	Antitumour, anti-inflammatory, neuroprotective, anxiolytic, osteogenic, cardiovascular protective, antimicrobial, antiparasitic [58,59]
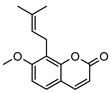				
244.1099				
**16** angelicin (isopsoralen)	*G. parviflora* (leaves)	GC-MS	[22]	Anti-cancer, pro-osteogenic, antiviral, pro-chondrogenic, anti-inflammatory, erythroid differentiating, anti-periodontitis [60,61]
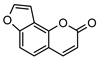				
186.0317				
**17** xanthyletine	*G. parviflora* (leaves)	GC-MS	[22]	Antimicrobial, fungicide [62,63]
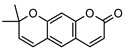				
228.0786				
**18** luvangetin	*G. balansae* (leaves)	UV, IR, ^1^H NMR, MS	[28]	Antiulcer, antifungal, anti-inflammatory, antibacterial [64,65]
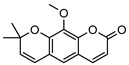				
258.0892				
**19** xanthoxyletin	*G. balansae* (bark)	UV, IR, ^1^H NMR, MS	[28]	Anticonvulsant, anti-inflammatory, carbonic anhydrase inhibitor, anti-malaria, histone lysine methyltransferase G9a inhibitor [66,67]
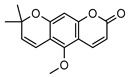				
258.0892				

(Key: *G.p*—*G. parviflora*, *G.s*—*G. salicifolia*).

### 3.2. Alkaloids

The isolation of twenty-two alkaloids has been reported from the genus *Geijera*, which includes five anthranilic acid derivatives, sixteen quinolones/quinolines (also derived from anthranilic acid), and a phenylethylamine-derived proto alkaloid hordenine. The five anthranilic acid derivatives **20–24** were isolated from the leaves of *G. parviflora* [68]. Three furoquinolines **25–27** [28,69], two isopropyldihydrofuroquinolines **28–29** [28,69], eight quinolones **30–37** [10,28,44], two dihydropyranoquinolines, **38–39** [27] and one dimeric quinolone **40** [28] have been isolated from the leaves, bark, and wood of species of *Geijera*. The quinolone flindersine **30** was isolated from the seeds/fruits of *G. parviflora* as well as from the leaves of *G. balansae* [28,44]. Additionally, hordenine **41** was isolated from the leaves of *G. balansae* [28] (Table 3).

Anthranilic acid derivatives are widely distributed within the *Rutaceae* family, but are of restricted distribution outside of this plant family [70]. The known physiological roles of alkaloids are generally accepted as providing protection for plants from pathogens and predators through their toxicity, as well as being involved in cell signalling and regulation of plant growth [71]. Alkaloids of genus *Geijera* display antimicrobial (including some against drug-resistant strains), anti-inflammatory, and other specific pharmacological activities as summarised in Table 3. Alkaloids, such as flindersine **30** and its derivatives, display significant activity in the mediation of inflammation, and these properties could help to explain the customary use of *G. parviflora* [10]. The dimeric quinolone alkaloid geijedimerine **40** from the leaves of *G. balansae* is unique to this species and has not been found so far in other taxa.

**Table 3:** Alkaloids identified within the genus *Geijera*.

Compound and Exact Mass (Da)	Source	Method of Identification	Reference	Pharmacological Activity of Compound (Various Sources)
**20** 11′-hexadecenoyl anthranilic acid	*G. parviflora* (leaves)	HRESI-MS, IR, UV, ^13^C and ^1^H NMR	[68]	Antibacterial vs. Gram positive bacteria [68]
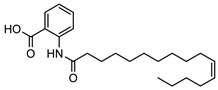				
373.2617				
**21** 9′-hexadecenoyl anthranilic acid	*G. parviflora* (leaves)	HRESI-MS, IR, UV, ^13^C and ^1^H NMR	[68]	Antibacterial vs. Gram positive bacteria [68]
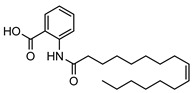				
373.2617				
**22** 7′-hexadecenoyl anthranilic acid	*G. parviflora* (leaves)	HRESI-MS, IR, UV, ^13^C and ^1^H NMR	[68]	Antibacterial vs. Gram positive bacteria [68]
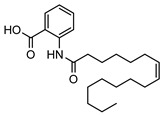				
373.2617				
**23** 9,12,15-octadecatrienoyl anthranilic acid	*G. parviflora* (leaves)	HRESI-MS, IR, UV, ^13^C and ^1^H NMR	[68]	Did not display significant antibacterial activity [68]
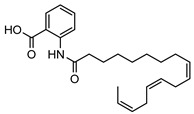				
383.2460				
**24** hexadecanoyl anthranilic acid	*G. parviflora* (leaves)	HRESI-MS, IR, UV, ^13^C and ^1^H NMR	[68]	Antibacterial vs. Gram positive bacteria [68]
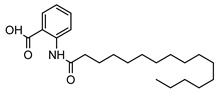				
375.2773				
**25** dictamnine	*G. balansae* (wood/bark)	^1^H NMR, IR, UV, and MS	[28]	Antibacterial, antiviral, antifungal, antiprotozoal, anti-cancer, anti-inflammatory, antioxidant, cardiovascular, antiplatelet, antiosteoporosis, anti-anaphylactoid [72]
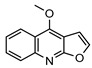				
199.0633				
**26** skimmianine	*G. salicifolia* (leaves)	IR, melting point (*G.s*)	[28,69]	Anti-inflammatory, acetylcholinesterase inhibitor, anti-cancer [73,74,75]
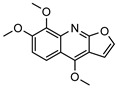	*G. balansae* (wood/bark)	^1^H NMR, IR, UV, and MS (*G.b*)		
259.0845				
**27** γ-fagarine	*G. salicifolia* (leaves)	IR, melting point (*G.s*)	[28,69]	Antileishmanial [76]
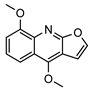	*G. balansae* (wood/bark)	^1^H NMR, IR, UV, and MS (*G.b*)		
229.0739				
**28** platydesmine	*G. salicifolia* (leaves)	Melting point, combustion analysis, chemical degradation, IR, UV and ^1^H NMR (*G.s*)	[28,69]	Antifungal [77]
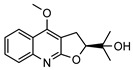				
259.1208	*G. balansae* (leaves)	^1^H NMR, IR, UV, and MS (*G.b*)		
**29** platydesmine acetate	*G. salicifolia* (leaves)	Combustion analysis, chemical degradation, IR and ^1^H NMR	[69]	No activity reported to date
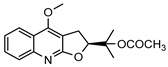				
301.1314				
**30** flindersine	*G. parviflora* (fruit/seeds)	IR and melting point (*G.p*)	[28,44]	Anti-inflammatory, collagen III suppression, antibacterial, antifungal [10,45,78]
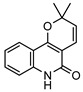	*G. balansae* (leaves)	^1^H NMR, IR, UV, and MS (*G.b*)		
227.0946				
**31** 4′-hydroxy-3′,4′-dihydroflindersine	*G. balansae* (leaves)	Chemical synthesis/derivatisation, ^1^H NMR, IR, UV, and MS	[28]	No activity reported to date
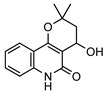				
245.1052				
**32** *cis*-3′,4′-dihydroxy-3′,4′-dihydroflindersine	*G. balansae* (leaves)	Chemical synthesis/derivatisation, ^1^H NMR, IR, UV, and MS	[28]	No activity reported to date
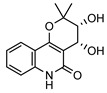				
261.1001				
**33** zanthobungeanine	*G. balansae* (leaves)	^1^H NMR, IR, UV, and MS	[28]	Leishmanicidal activity on Leishmania Viannia panamensis intracellular amastigotes (EC_50_: 8.7 µg/)mL and promastigotes (EC_50_: 14.3 µg/)mL, respectively [79]
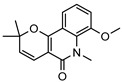				
271.1208				
**34** 8-(methoxyl)-flindersine	*G. parviflora* (leaves)	UV, IR, 2D NMR and MS	[10]	No activity reported to date
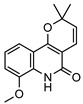				
257.1052				
**35** N-(acetoxymethyl) flindersine	*G. parviflora* (leaves)	UV, IR, 2D NMR and MS	[10]	Anti-inflammatory, collagen III suppression [10,45]
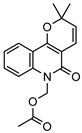				
299.1158				
**36** haplaphine	*G. parviflora* (leaves)	UV, IR, 2D NMR and MS (*G.p*)	[10,28]	Anti-inflammatory, cytotoxic [10]
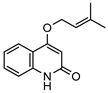	*G. balansae* (bark)	^1^H NMR, IR, UV, and MS (*G.b*)		
229.1103				
**37** 4-methoxy N-methyl-2-quinolone	*G. balansae* (bark)	^1^H NMR, IR, UV, and MS	[28]	Antimicrobial against MRSA, IC_50_ 8.0 µM [80]
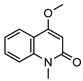				
189.0790				
**38** geibalansine	*G. balansae* (leaves)	Chemical synthesis/derivatisation, ^1^H NMR, IR, UV, and MS	[27]	Antispasmodic [81]
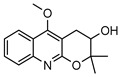				
259.1208				
**39** O-acetyl geibalansine	*G. balansae* (leaves)	Chemical derivatisation, ^1^H NMR, IR, UV, and MS	[27]	No activity reported to date
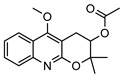				
301.1314				
**40** geijedimerine	*G. balansae* (leaves)	Chemical derivatisation, ^1^H NMR, IR, UV, and MS	[28]	No activity reported to date
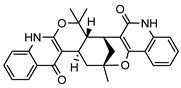				
454.1893				
**41** hordenine	*G. balansae* (leaves)	^1^H NMR, IR, UV, and MS	[27]	Diuretic, disinfectant, antihypotensive agent. Used for treatment of dysentery. Antifeedant for grasshoppers [67].
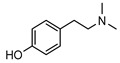				
165.1154				

(Key: *G.b*—*G. balansae*, *G.p*—*G. parviflora*, *G.s*—*G. salicifolia*).

### 3.3. Terpenes and Terpenoids

In total, sixty-four different terpenes/terpenoids have been isolated from the genus *Geijera* including monoterpenes, **42–55**, monoterpenoids, **56–66**, sesquiterpenes, **67–86**, sesquiterpenoids **87–104**, and the triterpene β-sitosterol **105**. Although many of the terpenes that have been reported within the *Geijera* species are minor constituents of the leaf essential oils, they have been included in this review due to the possibility that they might contribute to the overall biological activity displayed by the plant extracts through combined and/or synergistic action together with the other active constituents. It is evident that the unequivocal identification of several of the compounds reported via GC-MS analysis requires further characterisation using spectroscopic techniques to aid in the confirmation of their structures. This is especially important for the disambiguation of the structures of geometric isomers and stereoisomers that have been reported.

Initial investigations of the essential oils of *G. parviflora* conducted by Penfold determined the presence of at least two chemotypes; the first one (1) was dominated by the terpenes pinene (**49**, **50**) and camphene **51**, which constituted 80% of the essential oils; while the other (2) contained an abundance of brevifolin **106** (a phenolic ketone) and the sesquiterpene azulene **67** [17,18,19]. Azulene **67** has been isolated as part of both the leaf essential oils of *G. parviflora* and *G. salicifolia*, respectively. Azulene is unique as it is one of the few naturally occurring pigments that is blue in colour, and it is responsible for the deep blue colour of the leaf oil from its *G. parviflora* chemotype [16,18]. Brophy and Goldsack continued this research and identified a total of four *G. parviflora* chemotypes, the two previously identified by Penfold, together with (3) a chemotype in which the terpenoid linalool **61** and the sesquiterpenoid β-eudesmol **96** were dominant, and another (4) in which the sesquiterpenes pregeijerene **68**, geijerene **70**, and the terpenoid linalool **61** were the major constituents [16]. The brevifolin-dominated chemotype (2) also contained spathulenol **102**, globulol **92**, and viridiflorol **98** as major constituents, with a very small proportion of monoterpenes. However, since the sample subjected to investigation was a few years old, it is unclear if any of the volatiles/monoterpenes had been lost from the extract, and it is possible that the large proportion of spathulenol **102**, globulol **92**, and viridiflorol **98** could be artefacts formed by oxidation of bicyclogermacrene **77** [16].

Two chemotypes of *G. salicifolia* exist; one containing pinene (**49**, **50**), camphene **51**, and limonene **45** as the dominant compounds; while the second chemotype contains large amounts of the phenolic ketone, brevifolin **106**. Brevifolin **106** forms a large proportion of the essential oil of this chemotype and is obtained from the leaves via hydro-distillation [16]. *G. linearifolia* has not been found to exhibit different chemotypes, and its essential oils are dominated by spathulenol **102**, geranyl acetate **57**, bicyclogermacrene **77**, and (*E,E*)-farnesol **87** [16]. There is scope for characterisation of the terpenes from *G. balansae*, which has been neglected because the studies performed on this species only targeted the isolation of alkaloids.

Terpenes and terpenoids are ubiquitous in the plant kingdom and they form the most diverse and abundant classes of secondary metabolites found in nature. They exhibit a large variety of pharmacological activities such as anti-microbial, anti-inflammatory, neuroactive, psychoactive, anti-cancer, anti-oxidant, and pest resistance, as well as several other activities [82]. This is also reflected in the range of activities displayed by the terpenes/terpenoids that have been identified within the *Geijera* species.

**Monoterpenes**: To date, fourteen monoterpenes **42–55** have been identified from the *Geijera* species, all of which have been identified in the leaf essential oils of the plants (Table 4).

**Monoterpenoids**: Six acyclic monoterpenoids **56–61**, and five cyclic monoterpenoids **62–66**, have been identified as part of the leaf essential oils (Table 4).

**Sesquiterpenes**: Seven cyclic sesquiterpene **68**, **70**, **75**, **76**, **80–82**, ten bicyclic sesquiterpenes **67**, **69**, **72–74**, **77–79**, **83–84,** two tricyclic sesquiterpene **71**, and **85**, as well as one open-chain sesquiterpene, **86,** have been isolated as part of the leaf essential oils (Table 4).

**Sesquiterpenoids**: Two acyclic sesquiterpenoids **87**, **99**, one cyclic sesquiterpenoid **89**, seven bicyclic sesquiterpenoids **88**, **94–97**, **101**, **103** and eight tricyclic sesquiterpenoids **90–93**, **98**, **100**, **102** and **104** have been isolated from the leaves of *G. parviflora*, *G. salicifolia* and *G. linearifolia* (Table 4).

**Triterpene**: One triterpene, β-sitosterol **105** was isolated from the leaves of *G. salicifolia* (Table 4).

**Table 4:** Terpenes and terpenoids identified within the genus *Geijera*.

Compound and Exact Mass (Da)	Source	Method of Identification	Reference	Pharmacological Activity of Compound (Various Sources)
**42** (*E*)-β-ocimene	*G. linearifolia* (leaves) *G. salicifolia* (leaves) *G. parviflora* (leaves)	GC-MS	[16]	Anticonvulsant, antifungal, antitumour, plant pest resistance and attraction of plant pollinators (semiochemical) [83]
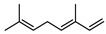				
136.1252				
**43** (*Z*)-β-ocimene	*G. linearifolia* (leaves) *G. salicifolia* (leaves) *G. parviflora* (leaves)	GC-MS	[16]	Anticonvulsant, antifungal, antitumour, plant pest resistance and attraction of plant pollinators (semiochemical) [83]
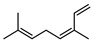				
136.1252				
**44** myrcene	*G. parviflora* (leaves) *G. salicifolia* (leaves)	GC-MS	[16]	Sedative, muscle relaxant, anti-inflammatory, analgesic, anti-tumour, antioxidant, psychotropic, antibiotic, antimutagenic [84,85]
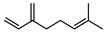				
136.1252				
**45** limonene	*G. salicifolia* (leaves)	GC-MS	[16]	Anxiolytic, anti-carcinogenic [84]
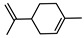				
136.1252				
**46** α-terpinene	*G. parviflora* (leaves) *G. salicifolia* (leaves)	GC-MS	[16]	Antioxidant, antimicrobial, acetylcholinesterase inhibition, sedative [85,86]
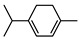				
136.1252				
**47** γ-terpinene	*G. parviflora* (leaves) *G. salicifolia* (leaves)	GC-MS	[16]	Antioxidant, antimicrobial, acetylcholinesterase inhibition, antinociceptive, anti-inflammatory [86,87,88]
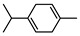				
136.1252				
**48** terpinolene	*G. parviflora* (leaves) *G. salicifolia* (leaves)	GC-MS	[16]	Antioxidant, antimicrobial, larvicide, insecticide [86,89]
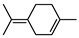				
136.1252				
**49** α-pinene	*G. parviflora* (leaves) *G. salicifolia* (leaves)	Chemical derivatisation (*G.p*) GC-MS (*G.s*)	[16,18]	Anti-inflammatory, anti-tumour [84]
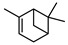				
136.1252				
**50** β-pinene	*G. parviflora* (leaves) *G. salicifolia* (leaves)	GC-MS	[16]	Anti-inflammatory, anti-tumour [84]
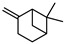				
136.1252				
**51** camphene	*G. parviflora* (leaves)	Chemical derivatisation	[18]	Antioxidant [90]
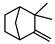				
136.1252				
**52** sabinene	*G. parviflora* (leaves) *G. salicifolia* (leaves)	GC-MS	[16]	Antioxidant, anti-inflammatory [91,92]
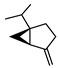				
136.1252				
**53** α-phellandrene	*G. parviflora* (leaves)	GC-MS	[16]	Antinociceptive, hyperthermic, promotes immune response, anti-cancer, antimicrobial, fungicide, pesticide [93]
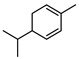				
136.1252				
**54** β-phellandrene	*G. parviflora* (leaves) *G. salicifolia* (leaves)	GC-MS	[16]	Acetylcholinesterase inhibitor, antifungal, expectorant [94,95]
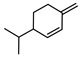				
136.1252				
**55** p-cymene	*G. salicifolia* (leaves) *G. parviflora* (leaves)	GC-MS	[16]	Antioxidant, anti-inflammatory, anti-cancer, antimicrobial [96]
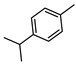				
134.1096				
**56** citronellyl acetate	*G. linearifolia* (leaves)	GC-MS	[16]	Pro-apoptotic activity in HepG2, fungicide, larvicide, bactericide, insect repellent/insecticide, antinociceptive [97]
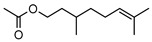				
196.1620				
**57** geranyl acetate	*G. linearifolia* (leaves)	GC-MS	[16]	Anti-cancer, antifungal [98,99]
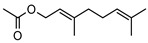				
196.1463				
**58** neryl acetate	*G. linearifolia* (leaves)	GC-MS	[16]	Fragrance and flavouring agent, strengthens skin barrier function [67,100]
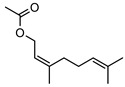				
196.1463				
**59** nerol	*G. linearifolia* (leaves)	GC-MS	[16]	Antimicrobial [101]
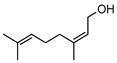				
154.1358				
**60** geraniol	*G. linearifolia* (leaves)	GC-MS	[16]	Antimicrobial [101]
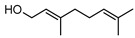				
154.1358				
**61** linalool	*G. linearifolia* (leaves) *G. salicifolia* (leaves) *G. parviflora* (leaves)	GC-MS	[16]	Anxiolytic, antibacterial, anti-inflammatory [102,103,104]
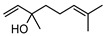				
154.1358				
**62** α-terpineol	*G. parviflora* (leaves) *G. salicifolia* (leaves)	GC-MS	[16]	Antioxidant, anti-cancer, anticonvulsant, antiulcer, antihypertensive, antinociceptive, enhances skin penetration, insecticidal properties [105]
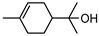				
154.1358				
**63** terpinen-4-ol	*G. parviflora* (leaves) *G. salicifolia* (leaves)	GC-MS	[16]	Anti-inflammatory, antifungal, anti-cancer, antibacterial [106,107,108,109,110]
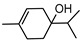				
154.1358				
**64** 1,8-cineole (eucalyptol)	*G. parviflora* (leaves) *G. salicifolia* (leaves)	GC-MS	[16]	Anti-inflammatory, antioxidant, analgesic, antifungal [107,111,112]
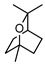				
154.1358				
**65** camphor	*G. parviflora* (leaves) *G. salicifolia* (leaves)	GC-MS	[22]	Insecticidal, antimicrobial, antiviral, anticoccidial, antinociceptive, anti-cancer, antitussive, skin penetration enhancer [113]
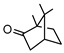				
152.1201				
**66** borneol	*G. salicifolia* (leaves)	GC-MS	[22]	Enhances membrane permeability, antibacterial, antifungal, antispasmodic, choleretic, acesodyne, sedative [114,115]
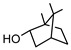				
154.1358				
**67** azulene	*G. parviflora* (leaves)	Chemical derivatisation	[18]	Anti-inflammatory [116]
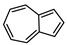				
128.0626				
**68** pregeijerene	*G. salicifolia* (leaves) *G. parviflora* (leaves)	Chemical derivatisation, degradative analysis, and UV	[29]	Antifeedant, oviposition deterrence [117]
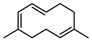				
162.1409				
**69** cogeijerene	*G. salicifolia* (leaves)	Chemical derivatisation, degradative analysis, and UV (*G.s*)	[29,118]	No activity reported to date
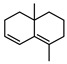				
162.1409	*G. parviflora* (leaves)	Chemical derivatisation, degradative analysis, IR, and UV (*G.p*)		
**70** geijerene	*G. parviflora* (leaves)	Combustion analysis, chemical derivatisation, degradative analysis, IR (*G.p*)	[16,18,119]	Antifeedant, oviposition deterrence [117]
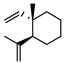				
164.1565	*G. salicifolia* (leaves)	GC-MS (*G.s*)		
**71** viridiflorene (ledene)	*G. linearifolia* (leaves)	GC-MS	[16]	Antifungal [120]
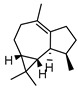				
204.1878				
**72** α-selinene	*G. parviflora* (leaves)	GC-MS	[16]	No activity reported to date
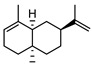				
204.1878				
**73** β-selinene	*G. parviflora* (leaves)	GC-MS	[16]	No activity reported to date
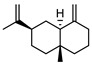				
204.1878				
**74** selina-3, 7(11)-diene	*G. parviflora* (leaves)	GC-MS	[22]	No activity reported to date
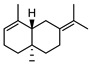				
204.1878				
**75** germacrene B	*G. salicifolia* (leaves)	GC-MS	[22]	Antimicrobial activity against Gram negative bacteria [121]
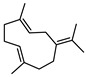				
204.1878				
**76** germacrene D	*G. linearifolia* (leaves) *G. salicifolia* (leaves) *G. parviflora* (leaves)	GC-MS	[16,22]	Anti proliferative, scavenging activity towards the ABTS radical, antibacterial, antifungal, insecticidal, repels herbivores, attracts pollinators [122,123]
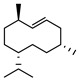				
208.2191				
**77** bicyclogermacrene	*G. linearifolia* (leaves) *G. salicifolia* (leaves) *G. parviflora* (leaves)	GC-MS	[16]	Larvicidal activity [124]
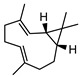				
204.1878				
**78** α-bergamotene	*G. parviflora* (leaves)	GC-MS	[16]	Antifeedant [125]
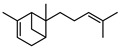				
204.1878				
**79** δ-cadinene	*G. parviflora* (leaves)	GC-MS	[16]	Acaricidal, antiproliferative and apoptotic [126,127]
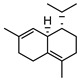				
204.1878				
**80** β-elemene	*G. linearifolia* (leaves) *G. salicifolia* (leaves) *G. parviflora* (leaves)	GC-MS	[16]	Anti-cancer, antineoplastic, reproductive toxicity [128,129]
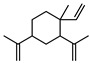				
204.1878				
**81** γ-elemene	*G. parviflora* (leaves) *G. salicifolia* (leaves)	GC-MS	[16]	Larvicidal activity [130]
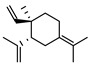				
204.1878				
**82** α-caryophyllene (humulene)	*G. salicifolia* (leaves)	GC-MS	[22]	Antibacterial, anti-inflammatory, antitumor, analgesic [131,132,133]
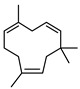				
204.1878				
**83** β-caryophyllene	*G. linearifolia* (leaves) *G. salicifolia* (leaves) *G. parviflora* (leaves)	GC-MS	[16]	Anti-inflammatory, analgesic, antimalarial, antifungal, antibacterial, anti-tumour [84,134]
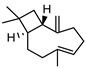				
204.1878				
**84** α-santalene	*G. parviflora* (leaves)	GC-MS	[22]	Insect repellent, semiochemical [125]
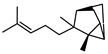				
204.1878				
**85** aromadendrene	*G. parviflora* (leaves) *G. linearifolia* (leaves) *G. salicifolia* (leaves)	GC-MS	[16,22]	Antibacterial (MRSA and drug resistant pathogens) [135]
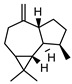				
204.1878				
**86** (*E,E*)-α-farnesene	*G. parviflora* (leaves) *G. linearifolia* (leaves) *G. salicifolia* (leaves)	GC-MS	[16]	Semiochemical, antibacterial, anticariogenic, anti-cancer, anti-plasmodial, hepatoprotective, antioxidant, anti-inflammatory, antifungal [136,137]
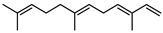				
204.1878				
**87** (*E,E*)-farnesol	*G. linearifolia* (leaves)	GC-MS	[16]	Antibacterial, antifungal [138,139]
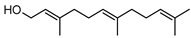				
222.1984				
**88** guaiol	*G. parviflora* (leaves) *G. salicifolia* (leaves)	GC-MS	[22]	Insecticide, antimicrobial, acaricidal, anti-cancer [140,141,142]
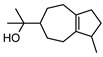				
208.1827				
**89** elemol	*G. parviflora* (leaves)	GC-MS	[16]	Antifungal [143]
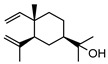	*G. salicifolia* (leaves)			
222.1984				
**90** palustrol	*G. linearifolia* (leaves)	GC-MS	[16]	Semiochemical [144]
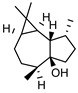				
222.1984				
**91** ledol	*G. parviflora* (leaves)	GC-MS	[22]	Antifungal, toxic CNS effects, antitussive, expectorant [145,146]
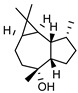				
222.1984				
**92** globulol	*G. parviflora* (leaves)	GC-MS	[16]	Antimicrobial [147]
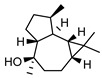				
222.1984				
**93** *epi*-globulol	*G. parviflora* (leaves)	GC-MS	[16]	Antimicrobial, semiochemical [148]
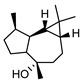				
222.1984				
**94** τ-cadinol	*G. linearifolia* (leaves)	GC-MS	[16]	Antitrypanosomal, smooth muscle relaxant, inhibits effects of cholera toxins [149,150]
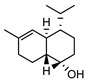				
207.1749				
**95** α-eudesmol	*G. linearifolia* (leaves) *G. parviflora* (leaves) *G. salicifolia* (leaves)	GC-MS	[16]	Antitrypanosomal, anti-cancer, anti-neurogenic inflammation [151,152,153]
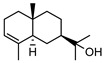				
222.1984				
**96** β-eudesmol	*G. linearifolia* (leaves) *G. parviflora* (leaves) *G. salicifolia* (leaves)	GC-MS	[16]	Anti-cancer, sedative, hepatoprotective, anti-inflammatory, diuretic, inhibits platelet aggregation, insect repellent, anti-allergy [67,152,154,155,156,157]
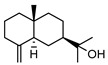				
222.1984				
**97** γ-eudesmol	*G. linearifolia* (leaves) *G. parviflora* (leaves) *G. salicifolia* (leaves)	GC-MS	[16]	Anti-cancer [152]
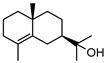				
222.1984				
**98** viridiflorol	*G. parviflora* (leaves)	GC-MS	[16]	Anti-mycobacterial, anti-inflammatory, antioxidant [158]
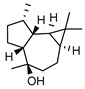				
222.1984				
**99** (*E,E*)-farnesal	*G. linearifolia* (leaves)	GC-MS	[16]	Semiochemical [159]
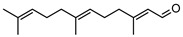				
220.1827				
**100** caryophyllene oxide	*G. linearifolia* (leaves) *G. salicifolia* (leaves) *G. parviflora* (leaves)	GC-MS	[16]	Anti-cancer, analgesic [134]
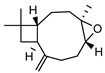				
220.1827				
**101** caryophylla-4(12), 8(13)-dien-5-ol	*G. parviflora* (leaves)	GC-MS	[22]	No activity reported to date
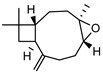				
220.1827				
**102** spathulenol	*G. linearifolia* (leaves) *G. parviflora* (leaves) *G. salicifolia* (leaves)	GC-MS	[16,22]	Antioxidant, anti-inflammatory, antiproliferative, antimycobacterial, antimicrobial [160,161]
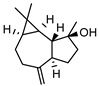				
220.1827				
**103** eremophilone	*G. parviflora* (leaves)	GC-MS	[16,22]	Cytotoxic, insecticidal, insect repellent, antifeedant (against termites) [162,163]
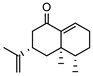				
218.1671				
**104** cyclocolorenone	*G. parviflora* (leaves)	GC-MS	[16,22]	Antifeedant, antimicrobial, allelopathic, anti-inflammatory, insect repellent [164]
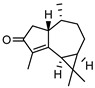				
218.1671				
**105** β-sitosterol	*G. salicifolia* (leaves)	Melting point and IR	[37]	Anti-cancer, anthelminthic, antimutagenic [165,166]
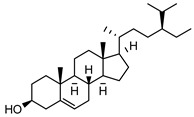				
414.3862				

(Key: *G.l*—*G. linearifolia*, *G.p*—*G. parviflora*, *G.s*—*G. salicifolia*).

### 3.4. Miscellaneous Compounds Isolated

Phenolic derivatives, brevifolin **106**, and elemicin **107**, have been identified within the leaf essential oils of *G. parviflora*. Brevifolin **106** also forms a large proportion of the essential oil from one chemotype of *G. salicifolia,* obtained from the leaves via hydro-distillation, and it is also present in the bark of *G. balansae*. A flavonoid 3,5,8,4′-tetrahydroxy-6,7-dimethoxyflavone **108**; a benzyl alcohol ester 2-phenylethyl isobutyrate **109**, fatty acid ester isoamyl isovalerate **110**, cyclic ketone *cis*-jasmone **111**, phenylpropanoid methyl eugenol **112**; and a benzene dicarboxylic acid (phthalic acid) **113** were also isolated from the leaves of *G. parviflora*. Additionally, four phenolic compounds **114–117** (vanillin, methyl syringate, methyl and ethyl ferulates, respectively) were isolated from the wood of *G. balansae* (Table 5).

The miscellaneous compounds isolated from the *Geijera* species exhibit a variety of pharmacological activities, as summarised in Table 5.

**Table 5:** Miscellaneous compounds isolated from the genus *Geijera*.

Compound and Exact Mass (Da)	Source	Method of Identification	Reference	Pharmacological Activity of Compound (Various Sources)
**106** brevifolin (xanthoxylin)	*G. parviflora* (leaves) *G. balansae* (bark) *G. salicifolia* (leaves)	GC-MS (*G.p*) ^1^H NMR, IR, UV, and MS (*G.b*) Melting point (*G.s*)	[16,18,28]	Antioxidant, hepatoprotective, antibacterial, antifungal, antinociceptive, antiedematogenic and antispasmodic [167,168]
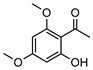				
196.0736				
**107** elemicin	*G. parviflora* (leaves)	GC-MS	[9]	Psychotropic, antimicrobial, antioxidant, acetylcholinesterase inhibitor, antiviral [9,169,170]
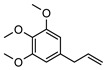				
208.1099				
**108** 3,5,8,4′-tetrahydroxy-6,7-dimethoxyflavone	*G. parviflora* (leaves)	^1^H and ^13^C NMR	[10]	No activity reported to date
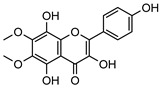				
346.0689				
**109** 2-phenylethyl isobutyrate	*G. parviflora* (leaves)	^1^H and ^13^C NMR	[10]	Odorant [171]
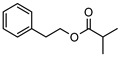				
192.1150				
**110** isoamyl isovalerate	*G. parviflora* (leaves)	^1^H and ^13^C NMR	[10]	Flavouring/odorant [172]
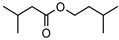				
172.1463				
**111** cis-jasmone	*G. parviflora* (leaves)	GC-MS	[22]	Semiochemical [173]
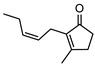				
164.1201				
**112** methyl eugenol	*G. parviflora* (leaves)	GC-MS	[22]	Attracts pollinator insects (semiochemical) [174]
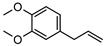				
178.0994				
**113** phthalic acid	*G. parviflora* (leaves)	GC-MS	[22]	Endocrine disruptor [175]
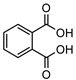				
166.0266				
**114** vanillin	*G. balansae* (wood)	^1^H NMR, IR, UV, and MS	[28]	Flavouring, pharmaceutical excipient, antioxidant, inhibits lipid peroxidation [67]
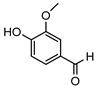				
152.0473				
**115** methyl syringate	*G. balansae* (wood)	^1^H NMR, IR, UV, and MS	[28]	Anti-diabetic, TRPA1 agonist [176,177]
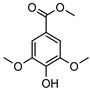				
212.0685				
**116** methyl ferulate	*G. balansae* (wood)	^1^H NMR, IR, UV, and MS	[28]	Inhibits COX-2 expression, blocks p-p38 and p-JNK in primary bone marrow derived-macrophages [178,179]
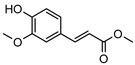				
208.0736				
**117** ethyl ferulate	*G. balansae* (wood)	^1^H NMR, IR, UV, and MS	[28]	Antioxidative, antiapoptotic, antirheumatic, neuroprotective and anti-inflammatory [180,181]
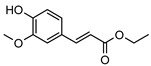				
222.0892				

(Key: *G.l*—*G. linearifolia*, *G.p*—*G. parviflora*, *G.s*—*G. salicifolia*).

## 4. Pharmacological Activities of *Geijera* Constituents

Compounds that have been identified within the genus *Geijera* exhibit a variety of pharmacological behaviors which can be categorised into the following main types of activity:


Antimicrobial activity;Antifungal activity;Reduction in inflammation;Reduction in pain;Reduction in anxiety;Muscle relaxant activity;Anti-cancer and anti-tumour activity;

Antioxidant activity;Acetylcholinesterase inhibition;MAO-B inhibition;Anticonvulsant activity;Psychotropic activity;Increase in membrane permeability;Plant pest resistance/insecticidal/semiochemical activity.


The chemical constituents identified in the four studied *Geijera* species are enumerated in Table 6, according to the main types of activity reported. The pharmacological activities of novel alkaloids isolated from *G. balansae*, namely O-acetyl geibalansine **39** and geijedimerine **40**, as well as the flindersine derivatives 4′-hydroxy-3′,4′-dihydroflindersine **31** and *cis*-3′,4′-dihydroxy-3′,4′-dihydroflindersine **32** are unknown, but in light of the activities reported from the other species of the genus, it would be worthwhile to examine these for any useful pharmacological properties.

The activities reported in Table 6 were obtained based on all the available literature for that chemical constituent. The purpose of this was to illustrate the range of pharmacological activities of these compounds, which can possibly support the customary uses of the plant.

### 4.1. Geijera Secondary Metabolites That Can Be Linked to Its Ethnobotanical Uses

The key pharmacological activities associated with the traditional use of *G. parviflora* are related to general analgesia, relief from toothache and infection, and the induction of psychoactive effects. These outcomes could arise from the following pharmacological activities as reported from specific secondary metabolites:

- anti-inflammatory activity
- analgesic/antinociceptive activity
- antimicrobial, antifungal, and antioxidant activity
- acetylcholinesterase inhibition, monoamine oxidase inhibition, muscle relaxant activity, sedative activity, anticonvulsant activity, and psychotropic activity (from neuro- and psycho-active compounds).

Although many of the active compounds identified are minor constituents, their combined activity (probable or possible synergistic activity) merits further investigation, in conjunction with the effects of compounds such as α-terpineol **62**, camphor **65**, and borneol **66**, which increase membrane permeability and hence may facilitate greater uptake of the active compounds. It has been hypothesised that the observed activities of preparations from medicinal plants can be attributed not only to the pharmacological effects of the main constituents, but also to a synergy of action between the most and the less abundant active components found within these mixtures [182]. The occurrence of a large variety of active major and minor constituents as observed within *G. parviflora*, makes it an ideal candidate for studies to explore the validity and implications of this hypothesis. In addition, many of the pharmacological activities herein are only reported for in vitro assays. This further supports the necessity for further investigations to ascertain the suitability of specific lead compounds for therapeutic use.

#### 4.1.1. Anti-Inflammatory, Analgesic, and Antinociceptive Compounds

Of the forty-four anti-inflammatory and analgesic compounds identified within the genus *Geijera*, thirty-four have been found in *G. parviflora* (Table 7). Since inflammation triggers cellular responses associated with pain and hyperalgesia, a decrease in inflammation should mitigate pain [183].

Several of the compounds in Table 7 display anti-inflammatory activity through the inhibition of inflammatory mediators. For example, caryophyllene oxide **100** was shown to inhibit cyclooxygenase and/or lipoxygenase, whereas compounds such as **13**, **26**, **30**, and **35**, act through the inhibition of nitric oxide and prostaglandin E_2_ production [57,73,92,106]. Banbury et al. suggested that the anti-inflammatory activities of flindersine **30** and its derivative (N-acetoxymethyl) flindersine **35**, which act through prostaglandin E_2_ inhibition, could contribute significantly to pharmacological effects that justify the traditional use of the leaves of *G. parviflora* for analgesia [10].

In total, nine compounds occurring in *G. parviflora* leaves: **44**, **47**, **53**, **62**, **64**, **65**, **83**, **100** and **106** have reported analgesic and/or antinociceptive activities, and these properties directly support the customary uses of this plant.

#### 4.1.2. Antimicrobial, Antifungal, and Antioxidant Compounds

A total of sixty-one antimicrobial, antifungal, and antioxidant compounds were identified within the *Geijera* species. These compounds (from various sources) have reported activities against a broad range of microbial and fungal pathogens, as well as significant antioxidant activities which may serve to support healthy immune responses and decrease the incidence of inflammatory conditions and resultant pain. Of these compounds, forty-one have been identified in *G. parviflora* (Table 8).

The furanocoumarin angelicin (isopsoralen) **16**, found in *G. parviflora* leaves, has reported activities against gamma-*herpes* viruses and periodontal disease [61,184], and these activities are congruent with the traditional use of the plant for toothache. Antimicrobial constituents such as hexadecanoyl anthranilic acid **24**, and the mixture of three anthranilic acid derivatives **20**, **21**, **22** from *G. parviflora* leaves displayed antibacterial activity against several Gram-positive strains, including a methicillin-resistant strain of *Staphylococcus aureus* [68]. Of particular interest, is that a quinolone isolated from the bark of *G. balansae*, 4-methoxy N-methyl-2-quinolone **37**, displays significant activity against Methicillin resistant *Staphylococcus aureus* (MRSA) with an IC_50_ value of 8.0 µM [80].

#### 4.1.3. Neuroactive and Psychoactive Compounds

The twenty-one compounds distributed within *Geijera* that display neuroactive and psychoactive effects are categorised in Table 9. In addition to these, the coumarin osthole **15** (from *G. parviflora* leaves) and the ferulic acid derivative ethyl ferulate **117** (from *G. balansae* wood) also possess neuroprotective properties [181,185]. A total of fifteen neuroactive and psychoactive compounds have been reported from *G. parviflora*.

In this group of compounds, geiparvarin **2** has been shown to be a strong and selective monoamine oxidase B inhibitor [43].

These constituents are present in minor quantities which may not be sufficient to produce psychoactive effects if taken orally (due to their metabolism in the digestive tract). However, the traditional use of *G. parviflora* for the purpose of inducing intoxication involves smoking the plant, and there may be high enough concentrations of actives (or pyrolysed actives) present within the smoke (which is absorbed directly into the bloodstream via the lungs) to induce intoxicating effects [22]. Preliminary investigation of smoke condensates from *G. parviflora* carried out by Sadgrove et al. did not yield definitive results [9]. Hence, there is scope for further work to be undertaken in order to refine the methodology devised to simulate the smoke preparations that are created during traditional use of *G. parviflora*, which are often produced in conjunction with other plant materials, so that any psychoactive constituents within these complex mixtures can be accurately determined and assayed for their combined activity, as well as their individual activities, in this context.

#### 4.1.4. Anti-Cancer Compounds

The most noteworthy anti-tumour compound isolated from the genus *Geijera* is geiparvarin **2**, which displays significant in vitro cytostatic activity and antiproliferative activity against various tumour cell lines [42,186]. The bioactivity of **2** was attributed to the furan-3 (2H) moiety, which was suggested by Borges et al. to act as an alkylating agent against bio-nucleophiles [41]. Geiparvarin **2** and 2′,3′-dihydrogeiparvarin **6** also display significant in vitro activity against human carcinoma of the nasopharynx [48,49]. Derivatives of geiparvarin **2** have been developed with increased cytotoxic activity, suggesting that this compound could provide a useful lead in the development of new anti-tumour agents [42].

In total, forty-one compounds displaying anti-cancer activities were reported within the genus *Geijera*, with thirty-three of these occurring in *G. parviflora* (Table 10). Although it is beyond the scope of this review to provide details of the various cancer cell lines that these compounds are active against, the number of compounds with anti-cancer activity present in *G. parviflora* especially, provides a good argument for the value and use of this plant in customary medicine.

#### 4.1.5. Compounds That Offer Pest Resistance, Insecticidal and Semiochemical Benefits

There are twenty-six compounds identified within the genus *Geijera* which have been observed in other studies to display useful botanical activities, including the ability to confer resistance from plant pests, provide protection from deleterious insects, and provide other semiochemical benefits such as anti-feedant activity and attraction of pollinators. Of these, twenty-one such compounds occur in *G. parviflora* (Table 11).

It would be useful to test extracts or isolates obtained directly from the *Geijera* species for the same or additional activities, such as antiparasitic activity. Based on the activities displayed here, there is scope for the development of formulations based on the constituents of *Geijera*, which could provide beneficial alternatives to conventional insect repellents as well as insecticides and pesticides in agricultural settings.

### 4.2. Future Perspectives

The traditional use of *G. parviflora* as an analgesic is supported by the identification of over thirty compounds within the plant, which display relevant pharmacological activities in this area. A promising range of active compounds has been discovered within other species of the genus, giving impetus for further natural product characterisation. Exploratory studies into synergistic effects are also warranted.

Most of the compounds identified within the genus *Geijera* have been isolated from the leaves of the plants. However, on the basis of the variety of active constituents that have been found within this species and its genus, it would be prudent to study the parts of the plant which have not received as much scientific attention, namely the fruits/seeds, which have previously yielded the alkaloid flindersine **30** [44].

The two New Caledonian species *G. cauliflora*, and *G. tartarea*, which have not been studied to date, should also be prioritised for future study.

Improvements in NMR and mass spectroscopy, and the development of new technologies for analytical separations and chemical profiling (LC/MS) have occurred in the decades since these studies were first performed. These advances mean that further compounds, including new structure derivatives, could be discovered. This could provide useful information in terms of the structure–activity relationships (SAR) of the currently known active compounds. In addition, a chemical profiling study that is focused on lead-like compounds, which compares the chemical profiles of different parts of the plants such as the leaves, fruits, and bark/wood, would also be beneficial to perform as an aid in further compound discovery. Further studies exploring a greater range of biological/physiological activities, beyond the traditional applications, are also worthwhile. This would include examining the agrochemical potential and bioactivity in a range of assays beyond those listed in this review, as well as exploration of the synthesis of active metabolites and/or their large-scale production, such as implementation of *callus* cultures. No studies have been conducted on the effect of the growing conditions on the production of secondary metabolites, nor has there been enough study of the plants in this genus to be able to establish whether specific compounds/classes observed could be chemotaxonomic markers. It is important to note that the pharmacological activities of the novel alkaloids O-acetyl geibalansine **39** and geijedimerine **40**, as well as the flindersine derivatives 4′-hydroxy-3′,4′-dihydroflindersine **31** and *cis*-3′,4′-dihydroxy-3′,4′-dihydroflindersine **32** isolated from *G. balansae* are unknown, but in the light of the activities reported from the other alkaloids of this genus, it would be helpful to examine these for useful pharmacological properties. This would include refining the methodology to extract these compounds, revisiting the complete characterisation of some of the compounds listed in this review, and exploring synthetic routes for their production.

## 5. Conclusions

Plants of the genus *Geijera* are a rich source of biologically active compounds which encompass terpenes, terpenoids, coumarins, quinolones, and anthranilic acid derivatives. The traditional use of *G. parviflora* in the Indigenous Australian context is supported by the presence of compounds with significant anti-inflammatory, analgesic, antioxidant, antimicrobial, and antifungal activity. The psychoactive, neuroactive, and neuroprotective aspects of constituents inferred from the traditional uses of *G. parviflora*, in conjunction with their reported activities, merit further detailed investigation. Studies undertaken in recent years have highlighted many of the biological activities of the chemical constituents within these plants including anti-cancer, antimicrobial, antifungal, and pest resistant properties. With such a wealth of bioactivity, compounds from the various species of *Geijera* still hold potential to provide new therapeutic agents. This justifies a thorough phytochemical investigation of the constituents of the two neglected species, *G. cauliflora*, and *G. tartarea*. Furthermore, based on the reported activities exhibited by their chemical constituents, additional research on the pharmacological potential of all the plant components, including the roots, stems, bark, leaves, and flowers, from the entire genus *Geijera* is justified.

## Figures and Tables

**Table 1:** *Geijera* plant species and their synonyms.

Species (Accepted Name)	Synonyms
*Geijera balansae* (Baill.) Schinz & Guillaumin	*Zanthoxylum balansae*
*Geijera cauliflora* Baill.	*Dendrosma deplanchei* Pancher & Sebert *Geijera deplanchei* (Pancher & Sebert) Däniker *Geijera lateriflora* Baill. ex Guillaumin
*Geijera linearifolia* (DC.) J.M.Black	*Geijera parviflora* var. crassifolia Benth. *Eriostemon linearifolius* DC. *Geijera linearifolia* Domin
*Geijera parviflora* Lindl.	*Geijera pendula* Lindl. *Geijera parviflora* var. *parviflora* Lindl. *Zanthoxylum australasicum* A.Juss.
*Geijera salicifolia* Schott	*Geijera salicifolia* var. *augustifolia* Maiden *Geijera salicifolia* Schott var. *salicifolia* *Geijera salicifolia* var. *latifolia* (Lindl.) Domin *Geijera salicifolia* var. *angustifolia* Maiden & Betche *Geijera latifolia* Lindl. *Geijera salicifolia* var. *typica* Domin *Geijera floribunda* Pancher ex Guillaumin
*Geijera tartarea* T.G.Hartley ex Munzinger & Bruy	None

**Table 6:** Constituents identified in *Geijera* species according to their pharmacological activity type.

Type of Activity	No. Compounds in *Geijera*	No. Compounds in *G. balansae*	No. Compounds in *G. parviflora*	No. Compounds in *G. salicifolia*	No. Compounds in *G. linearifolia*
Acetylcholinesterase inhibition	7	1	6	6	-
Anti-cancer	41	4	32	26	13
Anticonvulsant	4	1	3	3	2
Antifungal	25	5	16	13	9
Antimicrobial	45	9	29	19	12
Antioxidant	20	4	15	11	2
Increase in membrane permeability	3	-	2	3	-
Monoamine oxidase B inhibition	1	-	1	1	-
Muscle relaxant	5	2	2	3	1
Osteogenic	3	1	2	-	-
Plant pest resistance/semiochemical/ insecticide	26	1	21	14	9
Psychoactive	3	-	3	2	-
Reduction in anxiety	7	-	5	5	2
Reduction in inflammation	38	7	28	17	6
Reduction in pain	12	1	8	10	3

**Table 7:** Anti-inflammatory, analgesic, and antinociceptive compounds within the genus *Geijera*.

umbelliferone **1** S	xanthoxyletin **19** B	sabinene **52** P,S	β-caryophyllene **83** P,S,L
6′-dehydromarmin **5** P	dictamine **25** B	α-phellandrene **53** P	(*E*,*E*)-α-farnesene **86** P,S,L
(*R*)-6-O-(4-geranyloxy-2-hydroxy) cinnamoylmarmin **7** P	skimmianine **26** S,B	citronellyl acetate **56** L	α-eudesmol **95** P,S,L
parvifloranine A **8** P	flindersine **30** P,B	linalool **61** P,S,L	β-eudesmol **96** P,S,L
scoparone **11** P	N-(acetoxymethyl) flindersine **35** P	α-terpineol **62** P,S	viridiflorol **98** P
suberosin **12** P	haplaphine **36** P,B	terpinen-4-ol **63** P,S	caryophyllene oxide **100** P,S,L
dehydrogeijerin **13** P,S	myrcene **44** P,S	1,8 cineole **64** P,S	spathulenol **102** P,S,L
6-(methoxyl) geiparvarin **14** P	γ-terpinene **47** P,S	camphor **65** P,S	cyclocolorenone **104** P
osthole **15** P	α-pinene **49** P,S	borneol **66** S	brevifolin (xanthoxylin) **106** P,S,B
angelicin (isopsoralen) **16** P	β-pinene **50** P,S	azulene **67** P	methyl ferulate **116** B
luvangetin **18** B	p-cymene **55** P,S	α-caryophyllene (humulene) **82** S	ethyl ferulate **117** B

(Sources: P—*G. parviflora*, S—*G. salicifolia*, L—*G. linearifolia*, B—*G. balansae*).

**Table 8:** Antimicrobial, antifungal, and antioxidant compounds within the genus *Geijera*.

umbelliferone **1** S	zanthobungeanine **33** B	nerol **59** L	guaiol **88** P,S
auraptene **3** P	4-methoxy N-methyl-2-quinolone **37** B	geraniol **60** L	elemol **89** P,S
scoparone **11** P	hordenine **41** B	linalool **61** P,S,L	ledol **91** P
osthole **15** P	(*E*)-β-ocimene **42** P,S,L	α-terpineol **62** P,S	globulol **92** P
angelicin (isopsoralen) **16** P	(*Z*)-β-ocimene **43** P,S,L	terpinen-4-ol **63** P,S	*epi*-globulol **93** P
xanthyletine **17** P	myrcene **44** P,S	1,8 cineole **64** P,S	τ-cadinol **94** L
luvangetin **18** B	α-terpinene **46** P,S	camphor **65** P,S	α-eudesmol **95** P,S,L
xanthoxyletin **19** B	γ-terpinene **47** P,S	borneol **66** S	viridiflorol **98** P
11′-hexadecanoyl anthranillic acid **20** P	terpinolene **48** P,S	viridiflorene (ledene) **71** L	spathulenol **102** P,S,L
9′-hexadecenoyl anthranillic acid **21** P	camphene **51** P	germacrene B **75** S	cyclocolorenone **104** P
7′-hexadecanoyl anthranillic acid **22** P	sabinene **52** P,S	germacrene D **76** P,S,L	brevifolin (xanthoxylin) **106** P,S,B
hexadecanoyl anthranillic acid **24** P	α-phellandrene **53** P	α-caryophyllene (humulene) **82** S	elemicin **107** P
dictamnine **25** B	β-phellandrene **54** P,S	β-caryophyllene **83** P,S,L	ethyl ferulate **117** B
γ-fagarine **27** S,B	p-cymene **53** P,S	aromadendrene **85** P,S,L	
platydesmine **28** S,B	citronellyl acetate **54** L	(*E*,*E*)-α-farnesene **86** P,S,L	
flindersine **30** P,B	geranyl acetate **57** L	(*E,E*)-farnesol **87** L	

(Sources: P—*G. parviflora*, S—*G. salicifolia*, L—*G. linearifolia*, B—*G. balansae*).

**Table 9:** Neuroactive and psychoactive compounds within the genus *Geijera*.

Acetylcholinesterase Inhibitors	Anxiolytics and Sedatives	Muscle Relaxants and Anticonvulsants	Psychoactive Compounds
geijerin **10** P,S	osthole **15** P	xanthoxyletin **19** B	geiparvarin **2** P,S
dehydrogeijerin **13** P,S	myrcene **44** P,S	geibalansine **38** B	myrcene **44** P,S
skimmianine **26** S,B	limonene **45** S	(*E*)-β-ocimene **42** P,S,L	elemicin **107** P
α-terpinene **46** P,S	α-terpinene **46** P,S	(*Z*)-β-ocimene **43** P,S,L	
γ-terpinene **47** P,S	linalool **61** P,S,L	myrcene **44** P,S	
β-phellandrene **54** P,S	borneol **66** S	α-terpineol **62** P,S	
elemicin **107** P	β-eudesmol **96** P,S,L	borneol **66** S	
		τ-cadinol **94** L	
		brevifolin (xanthoxylin) **106** P,S	

(Sources: P—*G. parviflora*, S—*G. salicifolia*, L—*G. linearifolia*, B—*G. balansae*).

**Table 10:** Anti-cancer compounds within the genus *Geijera*.

umbelliferone **1** S	dictamnine **25** B	p-cymene **55** P,S	β-caryophyllene **83** P,S,L
geiparvarin **2** P,S	skimmianine **26** S, B	citronellyl acetate **56** L	(*E*,*E*)-α-farnesene **86** P,S,L
auraptene **3** P	haplaphine **36** P,B	geranyl acetate **57** L	guaiol **88** P,S
6′dehydromarmin **5** P	(*E*)-β-ocimene **42** P,S,L	α-terpineol **62** P,S	α-eudesmol **95** P,S,L
2′,3′-dihydrogeiparvarin **6** P,S	(*Z*)-β-ocimene **43** P,S,L	terpinen-4-ol **63** P,S	β-eudesmol **96** P,S,L
(R)-6-O-(4-geranyloxy-2-hydroxy) cinnamoylmarmin **7** P	myrcene **44** P,S	camphor **65** P,S	γ-eudesmol **97** P,S,L
scoparone **11** P	limonene **45** S	germacrene D **76** P,S,L	caryophyllene oxide **100** P,S,L
6-(methoxyl) geiparvarin **14** P	α-pinene **49** P,S	δ-cadinene **79** P	spathulenol **102** P,S,L
osthole **15** P	β-pinene **50** P,S	β-elemene **80** P,S,L	eremophilone **103** P
angelicin (isopsoralen) **16** P	α-phellandrene **53** P	α-caryophyllene (humulene) **82** S	β-sitosterol **105** S
xanthoxyletin **19** B			

(Sources: P—*G. parviflora*, S—*G. salicifolia*, L—*G. linearifolia*, B—*G. balansae*).

**Table 11:** Antifeedant, oviposition deterrent, insecticidal, and semiochemical compounds within the genus *Geijera*.

Insecticides	Semiochemicals	Antifeedants	Oviposition Deterrents
terpinolene **48** P,S	(*E*)-β-ocimene **42** P,S,L	umbelliferone **1** S	pregeijerene **68** S
α-phellandrene **53** P	(*Z*)-β-ocimene **43** P,S,L	hordenine **41** B	geijerene **70** S,P
citronellyl acetate **56** L	α-santalene **84** P	pregeijerene **68** S	
α-terpineol **62** P,S	(*E*,*E*)-α-farnesene **86** P,S,L	geijerene **70** S,P	
camphor **65** P,S	palustrol **90** L	α-bergamotene **78** P	
germacrene D **73** P,S,L	epi-globulol **93** P	eremophilone **103** P	
bicyclogermacrene **77** P,S,L	β-eudesmol **96** P,S,L	cyclocolorenone **104** P	
δ-cadinene **79** P	(*E*,*E*)-farnesal **99** L		
γ-elemene **81** P,S	*cis*-jasmone **111** P		
guaiol **88** P,S	methyl eugenol **112** P		
β-eudesmol **96** P,S,L			
eremophilone **103** P			

(Sources: P—*G. parviflora*, S—*G. salicifolia*, L—*G. linearifolia*, B—*G. balansae*).

## Data Availability

Not Applicable.
